# Effect of processing method of hybrid rye grain on growth performance, dietary net energy utilization, and carcass characteristics of yearling beef steers fed a finishing diet

**DOI:** 10.1093/tas/txaf102

**Published:** 2025-08-19

**Authors:** Federico Podversich, Warren C Rusche, Scott L Bird, Zachary K F Smith

**Affiliations:** Department of Animal Science, South Dakota State University, Brookings, SD 57007, USA; Department of Animal Science, South Dakota State University, Brookings, SD 57007, USA; Southeast Research Farm, South Dakota Agricultural Experiment Station, Beresford, SD 57004, USA; Department of Animal Science, South Dakota State University, Brookings, SD 57007, USA

**Keywords:** beef cattle, feedlot, grain processing, hybrid rye, winter cereal

## Abstract

This experiment evaluated the effects of replacing one-third of corn grain in a finishing diet with rye grain (RG) processed using one of three processing methods. Predominately Angus steers (n = 192, initial shrunk BW = 410 ± 20.9 kg) were blocked by source and pen location and assigned to one of four dietary treatments: dry-rolled corn (DRC), unprocessed RG (UNP), dry-rolled RG (DRR) and hammer-milled RG (HMR). Steers were fed for a total of 147 d. Pen was the experimental unit (6 pens per treatment, 8 steers per pen), and data were analyzed as a randomized complete block design using three contrasts: grain type [DRC vs. (UNP + DRR + HMR)], RG processing (UNP vs (DRR + HMR), and RG processing method (DRR vs HMR). Processing RG increased (*P* = 0.02) apparent neutral detergent fiber digestibility (aNDFD). Dry-rolling RG increased digestibility of dry matter and organic matter (*P *≤ 0.02) and tended (*P *= 0.09) to increase starch digestibility compared to HMR. Dry matter intake (DMI) was unaffected by grain type (*P* = 0.55) and whether RG was processed (*P* = 0.27), but processing method affected DMI (*P* < 0.01; 11.5 and 12.3 kg/d for DRR and HMR, respectively). Grain type did not affect (*P* = 0.18) gain to feed (G:F). Rye processing tended to increase G:F by 4.4% (*P* = 0.08), and DRR steers tended to be 4% more efficient than HMR steers (*P* = 0.10; 0.146 and 0.140, respectively). Observed Net Energy for gain (paNEg) tended to be 3% greater for DRC than steers fed RG (*P* = 0.09) with RG processing having no effect (P = 0.17). Steers fed DRR tended to have 4.5% greater paNEg than HMR steers (*P* = 0.06; 1.32 and 1.26 Mcal/kg, for DRR and HMR, respectively). Rye grain processing tended to decrease dressing percentage (*P *= 0.07) but no other effects on carcass characteristics or USDA grade distributions were observed (*P* ≥ 0.24). Liver abscess prevalence was unaffected by grain type (*P* = 0.81) and whether RG was processed (*P* = 0.77). However, processing method tended (*P *= 0.08) to influence liver abscess prevalence (78.4% and 91.8% normal livers for DRR and HMR, respectively). Rye grain effectively replaced one-third of DRC in a finishing diet with minor effects on performance or feed efficiency. Processing RG tended to improve efficiency, and using dry-rolling tended to improve feed efficiency compared to hammer-milling under the conditions of this experiment.

## Teaser

Rye can replace one-third of corn grain in feedlot diets with minor effects on growth and feed efficiency. Processing rye may improve feed efficiency with dry rolling resulting in greater net energy compared with hammer milling.

## INTRODUCTION

Cereal winter rye is a viable option for a rotational crop in North America (Rusche et al., 2020; Pereira et al., 2022; Zhang et al., 2024a). Feeding rye grain (RG) has been limited partly because of the presence of ergot alkaloids (EA), produced by the infection by Claviceps spp. (Coufal-Majewski et al., 2016; Zhang et al., 2024a). With the development of newer rye hybrids that have greater yield potential and increased resistance to ergot, interest in RG as feed for ruminants is growing (Rusche et al., 2020; Buckhaus et al., 2021; Zhang et al., 2024a). Additionally, feeding RG to ruminants could serve to utilize this cereal during periods of low demand in the milling and distillery industries. More information on the feed value of RG and the most effective strategies for processing and handling could enhance its use in beef cattle diets.

In previous studies at South Dakota State University evaluating RG, we demonstrated that RG could partially substitute for dry-rolled corn (DRC) in beef finishing diets (Rusche et al., 2020; Podversich et al., 2025). In those studies, substituting dry-rolled RG for one-third of DRC in the diet resulted in similar growth performance, feed efficiency, and carcass characteristics compared with when DRC was the sole grain source. This research provided assurance that cattle feeding could be a viable market, reducing the risk of incorporating hybrid rye into existing crop rotations. Nonetheless, this original study did highlight challenges with adopting RG.

Processing RG using the roller mill normally used for processing corn at that location was ineffective because of the size and shape of the rye kernels, necessitating a different roller mill to conduct that experiment (Rusche et al., 2020). In addition, we observed that replacing either two-thirds or 100% of the DRC with dry-rolled RG depressed dry matter intake (DMI) later in the feeding period (Rusche et al., 2020). Completely replacing DRC with unprocessed RG did not affect DMI, but did decrease gain and efficiency, likely because of reduced diet digestibility (Buckhaus et al., 2021).

Previous studies have evaluated different processing strategies for RG utilized in cattle diets on intake, nutrient digestibility, and ruminal fermentation parameters (Rajtar et al., 2020; Pereira et al., 2022). Rajtar et al. (2020), found that the starch in situ digestibility of whole unprocessed rye was only 10% and 20% after 8 and 24 hours of incubation, respectively, whereas crushing the rye grain increased digestibility up to approximately 90% at 8 hours of incubation. Pereira et al. (2022) observed that either fine or coarse dry rolling of rye grain results in similar total tract starch digestibility, approximately 98%, when this grain is fed to beef heifers. Tobin et al. (2023), replaced dry-rolled corn with rye grain processed in two different ways, rolled or ground, in a backgrounding diet where grains were included at 22% of the diet DM. Interestingly, in that study, rye-fed steers performed similarly than corn-fed steers, and ground-rye fed steers gained more and were more efficient than rolled-rye fed steers (Tobin et al., 2023).

Determining the effectiveness of grain processing options can influence acceptance of novel feedstuffs. If existing roller mills cannot be adjusted to appropriately reduce particle size it may be necessary to purchase or upgrade roller mills. This could represent a barrier to adoption if added investment is required to use a feedstuff. Using hammer mills (grinder mixers) offers the advantage of being widely accessible, but this approach can result in greater quantities of dust and fine particles (McKinney 2006), increasing acidosis risk. Alternatively, feeding a smaller inclusion rate of whole rye may be viable if the price difference between rye and corn was sufficient to offset possible efficiency losses.

The objective of this experiment was to compare differing processing methods when rye grain replaced approximately one-third of the corn. Our hypotheses were that: A) Replacing one-third of DRC with RG will result in similar growth performance and carcass outcomes of feedlot cattle, B) Processing RG would improve feed efficiency and carcass outcomes, and C) using either dry-rolled or hammer milled RG will lead to similar feed efficiency and carcass outcomes.

## MATERIALS AND METHODS

### Institutional Animal Care and Use Approval

The experiment was conducted at the Southeast Research Farm (SERF) in Beresford, SD, between March and August 2022. The animal care and handling procedures used in this study were approved by the South Dakota State University Animal Care and Use Committee (approval # 2202-010E).

### Dietary Treatments

Four treatments were used in a 147-d randomized complete block design experiment. The four treatments were: A) a control diet containing 60% DRC (DRC), and three treatments where rye was processed using three different methods and replaced approximately one-third of DRC, B) unprocessed rye (UNP), C) dry-rolled rye (DRR), or hammer-milled rye (HMR). All hybrid rye was produced at SERF in 2021. The DRR was rolled similarly to rye fed in our initial studies (Rusche et al., 2020) with a processing index of 78.7. The HMR was processed through a PTO-driven grinder-mixer with a 0.95 cm screen operated at 2000 rpm. This screen size was chosen to minimize the production of fines but sufficiently fracture rye kernels to enhance digestibility. Excess fines in processed small grains have been associated with decreased DMI (Zinn, 1993). Degree of processing has been shown to increase fermentation rate of RG (Ratjar et al., 2020; Pereira et al., 2022). Other researchers have observed adverse health effects in finishing cattle fed extensively processed RG (Wagner, 2022). Consequently, we elected to err on the side of caution to minimize the risk of acidosis-caused health issues by using a less aggressive screen.

Rye samples (processed and un-processed) were analyzed for particle size distribution and geometric mean diameter (GMD) at SGS North America, Inc. (Brookings, SD). Samples were split using a riffle splitter, and a 100-g subsample was weighed and sieved through a set of 13 circular sieves (3,350 μm; 2,380 µm; 1,680 μm; 1,190 μm; 841 μm; 595 μm; 420 μm; 297 µm; 210 μm; 149 μm; 105 μm; 74 μm; 53 μm; and pan) using a sieve shaker for 10 min. After the sample was shaken, the weight of the material on each sieve was recorded. No agitators or dispersion agents were used in the analysis.

Ergot alkaloids were determined by liquid chromatography—tandem mass spectrometry (North Dakota State University Veterinary Diagnostic Laboratory, Fargo, ND). The EA and particle size distributions for the hybrid rye used during this experiment are presented in Table 1. Processed rye grain (DRR and HMR) had numerically greater total EA concentrations compared to whole rye (615 ± 772.5, 3361 ± 1331.8, and 2527 ± 1727.5 ppb for UNP, DRR, and HMR, respectively). We suspect that those differences are related to sub-sampling and collection time. Sufficient quantities of DRR and HMR to complete the experiment were processed on a single day and stored in a covered commodity shed; UNP was removed from storage as needed over the course of the experiment. Consequently, UNP rye was sampled from a greater variety of locations within the storage bin. Using the weighted averages of RG inclusion from d22 to d 147, the dietary EA concentrations were 114, 615, and 469 ppb for UNP, DRR, and HMR, respectively, which are less than the maximum allowable EA concentration of 2,000 ppb for cattle diets (Coufal-Majewski et al., 2016).

**Table 1.** Ergot alkaloid content and particle size distribution for the rye grain from different processing methods

	Rye grain		
	Whole-unprocessed	Dry-rolled	Hammer-milled
*Ergot alkaloid concentration* ^1^ *, ppb*			
Ergocornine	77 ± 130.9	401 ± 213.8	406 ± 383.5
Ergocorninine	26 ± 31.8	130 ± 63.6	117 ± 110.6
Ergocristine	78 ± 134.3	813 ± 500.7	584 ± 456.8
Ergocristinine	22 ± 23.8	164 ± 116	101 ± 70
Ergocryptine	221 ± 367.3	872 ± 509	682 ± 733.1
Ergocryptinine	78 ± 170.2	242 ± 107.3	144 ± 137.1
Ergosine	64 ± 76.4	322 ± 218.8	190 ± 177.4
Ergosinine	26 ± 27.4	93 ± 58.3	58 ± 55.6
Ergotamine	30 ± 39.4	246 ± 148.5	200 ± 174.3
Ergotaminine	14 ± 9.2	79 ± 44.3	63 ± 55.9
Total Ergot alkaloid concentration	615 ± 772.5	3361 ± 1331.8	2537 ± 1727.5
*Screen size (μm), % retained*			
3,390	0.56	0.18	0.12
2,380	28.33	6.27	16.94
1,680	68.29	55.76	64.89
1,190	2.37	28.95	10.65
841	0.20	6.62	4.11
595	0.10	0.99	1.41
420	0.02	0.48	0.62
297	0.04	0.33	0.31
210	0.01	0.20	0.27
149	0.01	0.10	0.30
105	0.01	0.10	0.20
74	0.01	0.02	0.20
53	0.01	0.00	0.01
Fine particles (< 1,190)	0.41	8.84	7.43
GMD^2^, μm	2190 ± 1.3	1712 ± 1.3	1901 ± 1.4

^1^Ergot alkaloid content is presented as Mean ± Standard Deviation (n = 20 samples). Samples were analyzed by NDSU Veterinary Diagnostic Laboratory, Fargo, ND using liquid chromatography—tandem mass spectrometry.

^2^GMD: geometric mean diameter, presented as Mean ± Standard Deviation.

### Animals, Initial Processing, and Study Initiation

Predominately Angus steers (n = 192) with an initial bodyweight (BW) of 410 ± 20.9 kg were used in this study. Steers were purchased from two sources in eastern SD and transported to the SERF on February 28 and March 1^st^, 2022. On March 8^th^, steers were individually weighed for allotment purposes, identified with a unique individual ear tag, vaccinated against respiratory pathogens: infectious bovine rhinotracheitis, bovine viral diarrhea types 1 and 2, parainfluenza-3 virus, and bovine respiratory syncytial virus; Bovi-Shield Gold 5, Zoetis, Parsippany, NJ) and clostridial species (Ultrabac 7/Somubac, Zoetis), and administered pour-on moxidectin (Cydectin, Bayer, Shawnee Mission, KS). Steers were ranked within source by BW and allotted randomly to one of 24 open lot dirt pens (n = 8 steers/pen; 6 pens per treatment) so that there was a similar BW distribution within pens for each source, Pens were blocked by source and pen location within the feedyard, and the study initiated on March 10, 2022. Steers were administered a steroidal implant (200 mg trenbolone acetate and 28 mg estradiol benzoate; Synovex Plus, Zoetis) 28 d after study initiation.

### Diets and Intake Management

Steers were adapted to their final diet over a 21-d period using three step-up diets with the three processing types of rye included beginning on d 1 (March 10, 2022). Steers were fed once daily at 0800 hours, and bunks were managed according to a slick bunk management system (slick at 0800 h most mornings) to allow ad libitum access to feed with minimal day-to-day variation in feed deliveries. Feed was manufactured in a commercial mixer wagon (6.1 m^3^; Reel Auggie 3120, Kuhn North America, Inc., Brodhead, WI) with a scale resolution of 0.91 kg. The transition diets fed from d 0 to 21 are presented in Table 2. The final diets fed from d 22 to 147 are presented in Table 3. In the final diets, RG replaced 33.9, 33.5 and 33.8% of DRC for UNP, DRR, and HMR, respectively as a weighted average. Rye silage replaced corn silage and grass hay for the last 14 d because of feed supply (Table 3). Feed intake and diet formulations were summarized weekly. Steers that died or were removed from the study were assumed to have consumed feed equal to the pen mean DMI up to the point of death or removal. One steer from the DRC group was removed for reasons unrelated to dietary treatment. All data are reported on a deads and removals excluded basis.

**Table 2.** Composition of the transition diets (d 0 to 21) fed to the steers in each treatment^1^

	d 0 to 7				d 8 to 21			
	DRC	UNP	DRR	HMR	DRC	UNP	DRR	HMR
*Ingredient, % DM basis*								
Dry-rolled corn	35.0	23.3	23.3	23.3	45.0	30.0	30.1	30
Whole rye	--	11.7	--	--	--	15.4	--	--
Dry-rolled rye	--	--	11.7	--	--	--	15.1	--
Hammer-milled rye	--	--	--	11.8	--	--	--	15.4
MDGS^2^	19.8	19.8	19.8	19.8	19.7	19.6	19.6	19.5
Grass hay	18.1	18.1	18.1	18.1	9.9	9.8	9.9	9.9
Corn silage	23.2	23.2	23.2	23.1	21.5	21.3	21.4	21.3
Rye silage								
Liquid Supplement^3^	3.9	3.9	3.9	3.9	3.9	3.9	3.9	3.9
*Nutrient composition* ^4^								
DM, % as fed	58.5	58.7	58.6	58.6	58.1	58.4	58.2	58.4
OM, % DM	94.1	94	94	94	94.8	94.7	94.7	94.7
CP, % DM	14.3	14.9	15.0	15.0	14.1	15.0	15.0	15.0
NDF, % DM	31.4	32.1	32.1	32.1	26.4	27.1	27.2	27.1
Starch, % DM	31.8	29.9	30.0	30.0	38.6	36.5	36.4	36.5
NEm, Mcal/kg DM	1.86	1.83	1.83	1.83	1.95	1.90	1.90	1.90
NEg, Mcal/kg DM	1.20	1.17	1.17	1.17	1.28	1.24	1.24	1.24

^1^DRC: dry-rolled corn-based diet; or 1/3 replacement with rye grain as UNP: whole rye unprocessed, DRR: dry-rolled rye grain, or HMR: hammer-milled rye grain.

^2^MDGS: Modified distillers’ grains with solubles.

^3^Provided 33 g/1000 kg of monensin to the diet as well as vitamins and minerals to exceed requirements (NASEM, 2016).

^4^Dry matter was measured weekly, nutrient composition concentration were analyzed from composite ingredient samples. Net energy values were estimated from Preston (2017). Dry matter (DM), organic matter (OM), crude protein (CP), neutral detergent fiber (NDF), net energy for maintenance (NEm), net energy for gain (NEg).

**Table 3.** Composition of the finishing diets (d 22 to 147) fed to the steers in each treatment^1^

	d 22 to 133				d 134 to 147			
	DRC	UNP	DRR	HMR	DRC	UNP	DRR	HMR
*Ingredients, % DM basis*								
Dry-rolled corn	53.6	35.6	35.7	35.6	64.0	40.6	40.7	40.6
Whole rye	--	18.3	--	--	--	20.6	--	--
Dry-rolled rye	--	--	18.0	--	--	--	20.4	--
Hammer-milled rye	--	--	--	18.2	--	--	--	20.6
MDGS^2^	18.2	18.1	18.2	18.1	18.3	20.3	20.4	20.3
Grass hay	2.0	2.0	2.0	2.0	--	--	--	--
Corn silage	22.3	22.1	22.2	22.2	--	--	--	--
Rye silage	--	--	--	--	13.8	14.1	14.1	14.1
Liquid Supplement^3^	3.9	3.9	3.9	3.9	3.9	4.4	4.4	4.4
*Nutrient composition* ^4^								
DM, % fed	58.0	58.3	58.1	58.2	57.4	56.3	56.2	56.3
OM, % DM	95.5	95.3	95.4	95.4	95.0	94.6	94.6	94.6
CP, % DM	13.5	14.6	14.7	14.6	14.1	15.9	16.0	16.0
NDF, % DM	22.0	22.9	23.0	23.0	19.0	20.7	20.7	20.7
Starch, % DM	45.1	42.4	42.4	42.4	46.9	41.7	41.7	41.7
NEm, Mcal/kg DM	2.02	1.96	1.96	1.96	2.03	1.96	1.96	1.96
NEg, Mcal/kg DM	1.35	1.31	1.31	1.31	1.34	1.28	1.28	1.28

^1^DRC: dry-rolled corn-based diet; or 1/3 replacement with rye grain as UNP: whole rye unprocessed, DRR: dry-rolled rye grain, or HMR: hammer-milled rye grain.

^2^MDGS: Modified distillers’ grains with solubles.

^3^Provided 33 g/1000 kg of monensin to the diet as well as vitamins and minerals to exceed requirements (NASEM, 2016).

^4^Dry matter was measured weekly, nutrient composition concentration were analyzed from composite ingredient samples. Net energy values were estimated from Preston (2017). Dry matter (DM), organic matter (OM), crude protein (CP), neutral detergent fiber (NDF), net energy for maintenance (NEm), net energy for gain (NEg).

Actual diet formulations (Tables 2 and 3) were based upon weekly dry matter (DM) analysis (drying at 60 °C until no weight change was observed) and corresponding feed batching records. After weekly DM (method no. 935.29; AOAC, 2012), proximate analysis of each ingredient (except for liquid supplement) was conducted using weekly samples composited monthly. Crude protein (CP) was determined by first analyzing for N (method no. 968.06; AOAC, 2016; Rapid Max N Exceed; Elementar; Mt. Laurel, NJ, USA), and then multiplying the resulting value by 6.25. Ash was determined using AOAC method no. 942.05 (AOAC, 2012). Percentages of neutral detergent fiber (NDF) were assumed to be 9.7% and 15.4% for corn DRC and RG, respectively (NASEM, 2016). Analysis of NDF composition for all other ingredients was conducted as described by Goering and VanSoest, (1970) using heat-stable α-amylase and sodium sulfite as described by Van Soest et al. (1991) in an Ankom 200 Fiber Analyzer (Ankom Technology Corp.). Starch was analyzed using a commercially available kit (Megazyme Total Starch Assay Kit; AOAC method 996.11; AOAC, 1996). Dietary net energy (NE) values were derived from tabular energy values (Preston 2017).

### Digestibility Estimates

Estimation of the apparent nutrient digestibility was conducted based on Beck et al., (2023). Fecal samples were collected via rectal palpation on d 119, composited by pen, placed on ice and then frozen at −20 °C for later analysis. Diet samples were collected for the 3 d prior to fecal sampling to determine nutrient intake. Dry matter, organic matter (OM; DM less ash), NDF, and starch for both diet and fecal samples were analyzed using the same procedures as described for ingredients analysis. Indigestible NDF at 240 h was utilized as an internal marker, and the determination was analyzed by Dairyland Laboratories (Arcadia, WI).

### Cattle Management and Data Collection

Steers were weighed at the time of study initiation, d 28, 56, 84, 119, and the morning of study termination, d 147. Body weights were measured before the morning feeding, with a 4% pencil shrink applied to initial and final BW. After final BW determination, steers were shipped to Tyson Fresh Meats in Dakota City, NE and harvested the next day.

### Calculations

Average daily gain (ADG) was determined as the difference between final and initial shrunk BW divided by days on feed (147). Dry matter intake (DMI) was calculated from weekly feed delivered and dry matter measurements. Dry matter intake as a percentage of the body weight was calculated as DMI divided by the midpoint body weight during the study, multiplied by 100. Midpoint body weight was calculated as the average between the initial and final BW. The gain efficiency ratio (G:F) was calculated using ADG divided by DMI.

Estimated empty body fat (EBF) percentage and final BW at 28% EBF (AFBW) were calculated from observed carcass traits (Guiroy et al., 2002), and proportion of closely trimmed boneless retail cuts from carcass round, loin, rib, and chuck (Retail Yield, RY; Murphey et al., 1960). Performance-adjusted Net Energy (paNE) was calculated from daily energy gain (EG; Mcal/d): EG = (carcass-adjusted ADG from d 0 to 147)^1.097^ × 0.0557W^0.75^, where W is the mean equivalent shrunk BW [shrunk BW × (478/AFBW), kg; NRC, 1996] for the period from d 0 to 147. Maintenance energy required (EM; Mcal/d) was calculated by the following equation: EM = 0.077BW^0.75^ (Lofgreen and Garrett, 1968) where BW is the mean shrunk BW (using the average of carcass-adjusted final BW and BW from d 20). Using the estimates required for maintenance and gain the paNEm and paNEg values (Owens and Hicks, 2019) of the diet were generated using the quadratic formula: $x=\frac{-b\pm \sqrt{b^{2}-4ac}}{2c}$, where x = NEm, Mcal/kg, a = −0.41EM, b = 0.877EM + 0.41DMI + EG, c = −0.877DMI, and NEg was determined from: 0.877NEm—0.41 (Zinn and Shen, 1998; Zinn et al., 2008). Rye Net energy was calculated as follows: [(test diet NE—Control diet NE)/% rye inclusion] + Control diet NE.

Steers were visually matched to carcass identification at the slaughter facility by experienced personnel and hot carcass weight (HCW) was recorded as carcasses were weighed. Prevalence of abscessed livers and abscess severity were determined by a trained technician using the Elanco system as Normal (no abscesses), A- (1 or 2 small abscesses or abscess scars), A (2 to 4 well organized abscesses less than 1 inch diameter), or A + (1 or more large active abscesses greater than 1 inch diameter with inflammation of surrounding tissue). Video image data were obtained from the plant for ribeye area (REA), rib fat (RF), and USDA marbling scores. Marbling scores were used to determine USDA Quality Grade (QG) and Yield Grades (YG) were calculated according to the USDA regression equation (USDA, 2017) using HCW, video camera data for RF and REA and a plant specific algorithm to determine kidney, pelvic, and heart fat (KPH) percentage. Dressing percentage was calculated as HCW/(final BW × 0.96). Carcass adjusted (CA) final body weight was calculated as HCW/0.625. This adjustment factor was chosen as it matches benchmark data from cattle fed in the Upper Midwest (SD, MN, IA, and NE) in facilities where cattle were exposed outdoor environmental conditions (Dahlke et al., 2021).

### Statistical Analysis

Data were analyzed as a randomized complete block design using the GLIMMIX procedure of SAS 9.4 (SAS Inst. Inc., Cary, NC) with the pen as the experimental unit. The model included the fixed effect of dietary treatment and the random effect of block (cattle source and pen location with feedyard) where pen location within each block corresponded with batch fraction within each load of feed. For the distribution of categorical variables (distributions of USDA Yield and Quality grade, and liver score), counts for each category were entered by pen, and a multinomial analysis for ordinal data was conducted following the procedure recommended by Bowley (2015), using pen within treatment as the subject. The binomial variables of the proportion of cattle presenting liver scars or telangiectasia were analyzed using binomial distributions. Contrasts were used to determine the effects of A) Grain type = Corn vs. Rye [DRC vs. (UNP + DRR + HMR)], B) Rye processing = whole rye vs. processed [UNP vs. (DRR + HMR)], C) Processing type = dry-rolled vs. hammered milled (DRR vs. HMR). Differences were declared at *P* ≤ 0.05, and tendencies were considered when 0.10 > *P* > 0.05.

## RESULTS

Digestibility estimates are presented in Table 4. Grain type (corn vs. rye) did not affect any of the nutrient constituents analyzed (*P* ≥ 0.28). Processing of rye grain tended to decrease (*P* = 0.10) apparent NDF digestibility. Using a roller mill increased digestibility of DM and OM (*P* ≤ 0.02) and tended to increase starch digestibility (*P *= 0.09) under the conditions of the current experiment. Fecal starch determinations were 17.3, 20.8, 15.6, and 19.2% of the feces DM (pooled SEM = 2.50), for DRC, UNP, DRR, and HMR, respectively, with no contrasts being significant (Corn vs Rye *P* = 0.58; Rye Processing *P* = 0.15; Processor type *P* = 0.20). In the period immediately prior to fecal collection, steers fed HMR consumed greater quantities of DM compared to steers fed DRR (*P *< 0.01; 13.1 and 11.4 kg/d, respectively).

**Table 4.** Total tract digestibility estimates for finishing steers fed diets where hybrid rye grain with different extents of processing partially replaced dry-rolled corn (d119 of a 147-d finishing experiment)^1^

	Treatment^2^					*P*-value^3^		
Item^4^	DRC	UNP	DRR	HMR	SEM^5^	C vs. RG	Rye Proc.	Proc. Type
DMI, kg/d	12.2	12.8	11.4	13.1	0.40	0.65	0.20	<0.01
DMD, %	69.4	68.3	73.0	66.7	1.67	1.00	0.45	0.01
OMD, %	71.4	70.2	74.8	68.6	1.76	0.92	0.48	0.02
aNDFD^6^, %	52.5	56.4	54.3	48.8	2.23	0.80	0.10	0.11
StarchD, %	87.6	85.3	89.9	84.2	2.44	0.66	0.55	0.09

^1^Substitution of dry-rolled corn with rye during the sampling period was 34.1, 33.6, and 33.6% for UNP, DRR, and HMR, respectively. Total tract digestibility estimates determined as described by Beck et al., (2023).

^2^DRC: dry-rolled corn based diet; UNP: unprocessed whole rye replacing 34.1% of DRC; DRR: dry-rolled rye replacing 33.6% of DRC; HMR: hammer-milled rye replacing 33.6% of DRC.

^3^Observed significance for the contrasts: C. vs. RG. = Corn vs. Rye grain (DRC vs. UNP + DRR + HMR), Rye Proc. = Rye processing (UNP vs. DRR + HMR), Proc. Type = Processing type (DRR vs. HMR).

^4^DMI: dry matter intake; DMD: dry matter digestibility; OMD: organic matter digestibility; aNDFD: apparent neutral detergent fiber digestibility; StarchD: starch digestibility.

^5^Standard Error of the Mean (n = 6 pens/treatment).

^6^Analyzed using heat-stable α-amylase and sodium sulfite as described by Van Soest et al., (1991).

Growth performance data are presented in Table 5. Intake was unaffected by grain type (*P* = 0.55), and RG processing (*P* = 0.27). Conversely, RG processing method affected intake where DRR-fed steers consumed 6.9% less feed compared to HMR-fed steers (*P* ≤ 0.02; 11.5 and 12.3 kg/d, respectively). No differences were observed for final BW or ADG for any of the contrasts evaluated (*P* ≥ 0.26). Grain type did not affect G:F (*P* = 0.18). Feeding whole RG tended to reduce G:F by 4.4% compared with processed RG (*P* = 0.08). Steers fed DRR tended to be 4% more efficient compared with HMR-fed steers (*P* = 0.10; 0.146 vs. 0.140, respectively). Carcass adjusted final BW were unaffected by grain type, RG processing, or processing method (*P* ≥ 0.25). Performance adjusted NEm and NEg (paNEm and paNEg) tended to be 2.7 and 3% greater, respectively, for DRC compared with rye-fed steers (*P* = 0.09). Processing RG did not affect paNEm and paNEg (*P* = 0.17). Steers fed DRR tended to have 3 and 4.5% greater paNEm and paNEg, respectively, as compared with HMR-fed steers (*P* = 0.06). The observed to expected (O:E) ratios for NE values were unaffected by grain type (*P* ≥ 0.72) and RG processing (*P* ≥ 0.17). However, O:E ratios for NEm and NEg were greater for DRR-fed steers than for HMR-fed steers (*P *= 0.05). No differences were observed in the apparent NE of whole RG compared with processed RG (*P* = 0.16); however, rolling rye tended to increase NEm and NEg by 14.8 and 19.5%, respectively, compared with HMR (*P* = 0.06).

**Table 5.** Growth performance of finishing steers fed either a dry-rolled corn-based diet or diets with one-third replacement of corn grain by rye grain under different processing strategies^1^

	Treatment^2^					*P*-value^3^		
Item^4^	DRC	UNP	DRR	HMR	SEM^5^	C. vs RG	Rye Proc.	Proc. Type
Steers, n	48	48	48	48	.	.	.	.
Pens, n	6	6	6	6	.	.	.	.
Initial BW (d 0), kg	409	410	411	410	.	.	.	.
Final BW (FBW, d 147), kg	661	653	657	662	7.5	0.52	0.26	0.51
ADG^6^, kg	1.71	1.65	1.68	1.71	0.044	0.35	0.29	0.39
DMI, kg	11.8	12.1	11.5	12.3	0.18	0.55	0.27	< 0.01
DMI, % of BW	2.21	2.27	2.15	2.29	0.034	0.45	0.11	< 0.02
G:F^6^, kg/kg	0.145	0.137	0.146	0.140	0.0032	0.18	0.08	0.10
CA FBW^7^, kg	695	691	684	694	8.1	0.46	0.79	0.25
* Energetics* ^8^								
paNEm, Mcal/kg	1.98	1.90	1.97	1.91	0.028	0.09	0.17	0.06
paNEg, Mcal/kg	1.32	1.26	1.32	1.26	0.024	0.09	0.17	0.06
O:E^9^ NEm	0.98	0.98	1.01	0.98	0.014	0.72	0.17	0.05
O:E^9^NEg	0.99	0.97	1.02	0.98	0.019	0.94	0.17	0.05
Rye^10^ NEm	.	1.64	1.96	1.67	0.108	.	0.16	0.06
Rye^10^ NEg	.	1.05	1.33	1.07	0.094	.	0.16	0.06

^1^Substitution of dry-rolled corn with rye after transitioning to the final diet (d 22 to 147) was 33.9, 33.5, and 33.8% for UNP, DRR, and HMR, respectively.

^2^DRC: dry-rolled corn based diet; UNP: unprocessed whole rye replacing 33.9% of DRC; DRR: dry-rolled rye replacing 33.5% of DRC; HMR: hammer-milled rye replacing 33.8% of DRC.

^3^Observed significance for the contrasts: C. vs. RG. = Corn vs. Rye grain (DRC vs. UNP + DRR + HMR), Rye Proc. = Rye processing (UNP vs. DRR + HMR), Proc. Type = Processing type (DRR vs. HMR).

^4^BW: body weight, ADG: average daily gain, DMI: dry matter intake, G:F: gain to feed.

^5^Standard Error of the Mean (n = 6 pens/treatment).

^6^Calculated using FBW.

^7^Carcass Adjusted (CA) FBW calculated using hot carcass weight (HCW) adjusted to a common DP of 62.5%.

^8^Performance adjusted Net Energy values (for maintenance and gain) were calculated using adjusted final body weight (AFBW) as mature BW (Guiroy et al., 2002).

^9^Observed over expected Net Energy = performance apparent NE/dietary calculated NE.

^10^Rye NE (for maintenance and gain), Mcal/kg of diet DM = [(test diet NE—DRC diet NE)/Rye inclusion] + DRC diet NE.

Carcass data are presented in Table 6. Neither grain type nor RG processing affected HCW, REA, RF, Marbling score, calculated USDA YG, RY, EBF, or AFBW (*P* ≥ 0.21). Processing RG tended to increase dressing percentage (*P* = 0.07), while grain type and rye processing method did not affect this parameter (*P* ≥ 0.31). Distributions of USDA QG and YG were unaffected by grain type, RG processing, or processing method (*P* ≥ 0.19).

**Table 6.** Carcass characteristics of finishing steers fed either a dry-rolled corn-based diet or diets with one-third replacement of corn grain by rye grain under different processing strategies^1^

	Treatment^2^					*P*-value^3^		
Item	DRC	UNP	DRR	HMR	SEM^4^	C. vs RG	Rye Proc.	Proc. Type
HCW^5^, kg	434	432	428	434	5.1	0.46	0.79	0.25
DP^6^, %	65.7	66.2	65.0	65.6	0.39	0.80	0.07	0.31
REA^7^, sq.cm	89.2	88.0	88.3	89.8	1.62	0.70	0.48	0.36
Rib fat, cm	1.92	1.90	1.79	1.82	0.096	0.43	0.38	0.84
Marbling score^8^	573	564	538	558	15.9	0.24	0.36	0.34
Yield Grade^9^	3.97	3.99	3.84	3.83	0.154	0.60	0.35	0.98
KPH^10^, %	1.83	1.83	1.83	1.80	0.030	0.72	0.55	0.31
Retail Yield^11^, %	48.5	48.5	48.8	48.8	0.33	0.60	0.34	0.92
EBF^12^, %	34.7	34.6	33.7	34.0	0.60	0.38	0.32	0.70
AFBW^13^, kg	571	569	576	581	11.0	0.52	0.21	0.57
*Yield grade, %*								
1	-	-	-	-	.	0.56	0.36	0.90
2	4.5	4.4	6.6	6.2				
3	54.9	54.3	61.9	60.9				
4	36.7	37.3	28.9	30.0				
5	3.9	4.0	2.7	2.8				
* Quality grade, %*								
Select	4.2	5.9	8.0	5.2	.	0.19	0.75	0.25
Low Choice	20.3	25.8	31.2	23.8				
Average Choice	34.7	35.8	35.1	35.7				
High Choice	30.5	25.1	20.2	27.0				
Prime	10.3	7.4	5.4	8.3				

^1^Substitution of dry-rolled corn with rye after transitioning to the final diet (d 22 to 147) was 33.9, 33.5, and 33.8% for UNP, DRR, and HMR, respectively.

^2^DRC: dry-rolled corn based diet; UNP: unprocessed whole rye replacing 33.9% of DRC; DRR: dry-rolled rye replacing 33.5% of DRC; HMR: hammer-milled rye replacing 33.8% of DRC.

^3^Observed significance for the contrasts: C. vs. RG = Corn vs. Rye grain (DRC vs. UNP + DRR + HMR), Rye Proc. = Rye processing (UNP vs. DRR + HMR), Proc. Type = Processing type (DRR vs. HMR).

^4^Standard Error of the mean (n = 6 pens/treatment).

^5^HCW: Hot carcass weight.

^6^DP: Dressing percentage.

^7^REA: Ribeye area.

^8^400 = small^00^ = Low Choice.

^9^Calulated using HCW, REA, rib fat, and KPH.

^10^KPH: kidney, pelvic and heart fat.

^11^Estimated proportion of trimmed boneless retail cuts from carcass round, loin rib, and chuck (Murphey et al., 1960).

^12^EBF: Empty body fat, calculated based on Guiroy et al., 2002.

^13^Final BW, adjusted at 28% EBF based on Guiroy et al., 2002.

Liver abscess data are presented in Table 7. Liver abscess incidence and severity were unaffected by grain type (*P* ≥ 0.81) or by RG processing (*P* ≥ 0.77). Processing method tended to alter the distribution of liver abscesses (*P* = 0.08), where DRR-fed steers presented 78.4% normal livers as compared with HMR-fed steers with 91.8% normal livers. In addition, the proportion of severe liver abscesses was approximately 3X greater for DRR than HMR steers. Despite this, no differences were observed in the distribution of liver scars and telangiectasia (*P* > 0.26).

**Table 7.** Liver characteristics of finishing steers fed either a dry-rolled corn-based diet or diets with one-third replacement^1^ of corn grain by rye grain under different processing strategies^1^

	Treatment^2^					*P*-value^3^		
Item	DRC	UNP	DRR	HMR	SEM^4^	C. vs RG	Rye Proc.	Proc. Type
Liver score^5^, %								
Normal	85.7	88.2	78.4	91.8		0.81	0.77	0.08
A-	8.5	7.2	12.4	5.1				
A	1.8	1.5	2.8	1.0				
A + or greater	3.9	3.2	6.3	2.1				
Liver scars, %	26.2	22.0	16.6	19.9	6.71	0.34	0.60	0.69
Telangiectasia, %	13.8	8.2	8.5	8.2	5.26	0.26	0.98	0.97

^1^Substitution of dry-rolled corn with rye after transitioning to the final diet (d 22 to 147) was 33.9, 33.5, and 33.8% for UNP, DRR, and HMR, respectively.

^2^DRC: dry-rolled corn based diet; UNP: unprocessed whole rye replacing 33.9% of DRC; DRR: dry-rolled rye replacing 33.5% of DRC; HMR: hammer-milled rye replacing 33.8% of DRC.

^3^Observed significance for the contrasts: C. vs. RG = Corn vs. Rye grain (DRC vs. UNP + DRR + HMR), Rye Proc. = Rye processing (UNP vs. DRR + HMR), Proc. Type = Processing type (DRR vs. HMR).

^4^Standard Error of the mean (n = 6 pens/treatment).

^5^Determined according to the Elanco Liver Scoring System.

## DISCUSSION

Our approach when selecting processing methods for this experiment, particularly for the HMR treatment, was to be on the side of caution and minimize acidosis risk. Based on the particle size of the resulting grain, digestibility estimates derived from fecal samples taken during the feeding period, as well as growth performance measures, the HMR treatment was under-processed. We were successful in reducing the proportion of fine particles in HMR compared to DRC (< 1,100 µm) but hammer-milling was less effective than rolling in reducing GMD. Hammer milling rye grain more extensively might result in different results than those observed in the current experiment.

The differences in digestibility estimates observed in the current experiment between DRR and HMR are reflective of the differences in degree of grain processing. Our results are consistent with those observed with varying degrees of rye grain processing (Ratjar et al., 2020; Pereira et al., 2022. The differences we observed between DRR and HMR are confounded with degree of processing and DMI, as steers fed the less extensively processed grain also consumed greater quantities of feed, which may have influenced rate of passage and digestibility. Grain processing did not increase the digestibility of starch or OM; however, our results may be biased because of inadequate processing for HMR. The lack of differences between DRC and RG may also be unduly influenced by results observed for UNP and HMR. This experiment was not designed to fully explore the effects of rye processing on nutrient digestibility; therefore, these results should be interpreted with caution. Yet, the study by Beck et al. (2023) determined a Pearson correlation of 0.87 for apparent total tract digestibility of DM, between the estimates for total collection and one-time sampling using indigestible NDF as a marker.

In the current experiment, dry-rolling reduced DMI compared to using a hammer mill, while there were no differences from RG processing or grain type. The extent of kernel processing alters the rate of ruminal fermentation (Offner et al., 2003), which could partially explain the reduction in intake when feeding DRR compared with HMR. Differences in fermentation rate of starch could result in ruminal pH decreases, leading to sub-acute acidosis and reduced feed intake (Owens et al., 1998). Rapidly fermented grain sources and extensively processed grain can have excessive rates of acid production in the rumen, causing subclinical acidosis and leading to reductions in DMI (Fulton et al., 1979; Owens et al., 1997). Rye grain is rapidly degraded in the rumen, with starch degradation rates significantly greater when compared with triticale and barley (Krieg et al., 2017) or to dry-rolled or high-moisture corn (Rusche and Smith, 2024). Increased inclusion of rye grain has been reported to decrease both mean ruminal pH and duration of time that ruminal pH was < 5.5 (Zhang et al., 2024b). While the current experiment did not directly measure ruminal pH, differences in particle size caused by processing would be consistent with altered starch fermentation. In research evaluating increasing inclusions of steam-flaked rye grain replacing steam-flaked corn, greater incidence of acidosis caused by rapidly fermentable rye grain was implicated as a potential factor for reduced cattle performance (Wagner, 2022). Alternatively, reduced DMI for DRR may be in response to satiety signals caused by greater ruminal starch fermentation (Allen et al., 2009). This explanation is supported by the fact that in the current experiment growth rates did not differ between processing methods even with a reduction in DMI.

Ergot alkaloids also have been identified as a cause of depressed DMI and cattle performance when feeding RG, particularly when EA concentrations exceed 750 ppb (Sarich et al., 2023). The DRR finishing diet in the current experiment had numerically the greatest dietary concentration of EA and in fact the least DMI. However, EA concentration alone cannot explain why HMR steers consumed numerically greater quantities of feed compared with UNP despite a dietary EA concentration more than 4X greater. Nonetheless, EA do represent a risk factor for RG implementation and an additional reason for blending RG with other grain sources in finishing cattle diets.

To the best of our knowledge, this is the first study to determine the effects of dry rolling compared to hammer milling for RG on growth performance and feed efficiency of finishing cattle. In agreement with our previous studies (Rusche et al., 2020; Podversich et al., 2025), replacing one-third of DRC with dry-rolled RG, or approximately 18% to 20% of the diet DM, did not affect growth performance. The current experiment lends support to the conclusion that a blend of one-third of dry-rolled RG and two-thirds of corn grain can be a helpful strategy to include RG in finishing diets without compromising performance. Our results are consistent with results from replacing barley with hybrid rye in finishing cattle diets, where limiting rye inclusion to 33% of the barley grain was the optimal rye inclusion strategy (Zhang et al., 2024a).

The observed tendency for reduced efficiency in the steers fed whole RG compared with steers fed processed RG supports the conclusion that grain processing increases nutrient utilization and enhances feed efficiency (Owens and Zinn 2005; NASEM, 2016) and is consistent with results where whole RG completely replaces DRC (Buckhaus et al., 2021). Because of similar ADG growth with reduced DMI, steers fed DRR in the current experiment tended to be more efficient than HMR-fed steers. In contrast, Tobin et al. (2023) fed backgrounding steers a high forage diet containing 22.1% RG, either dry-rolled or hammer milled, and observed improved ADG and GF with the hammer milled rye (Tobin et al., 2023). In that study, researchers used a smaller screen compared with the current experiment ((0.64 vs. 0.95 cm, C. Tobin, personal communication). Their results are not completely applicable as more aggressive grain processing in a diet with greater concentrations of roughage would be less likely to cause sub-acute acidosis and may be beneficial if greater starch digestibility caused increased energy capture without reducing NDF digestibility. However, their results combined with the differences in GMD and starch digestibility observed in the current experiment between the two processed RG support the conclusion that the HMR in the current study was insufficiently processed to optimize cattle performance. Less severe processing of wheat and barley has been associated with poorer feed efficiency (Jancewicz et al., 2017) and reduced corn grain particle size led to decreased DMI in finishing cattle, like the responses observed in the current experiment (Schwandt et al., 2016).

We observed that the O:E ratio for NE values was increased in DRR compared to HMR and was numerically greater than the DRC control. These results suggest that there may be a positive associative effect to including approximately 20% DRR in diet DM combined with DRC, similar to what we observed in our earlier work (Rusche et al., 2020). Previous research suggested that blends between dry-rolled corn and cereal grains can improve the growth performance of beef cattle (Kreikemeier et al., 1987). Kreikemeier et al. (1987) observed improved ADG and GF by 4% and 4.4%, when steers were fed mixtures of DRC and wheat grain, at a ratio of one-third and two-thirds of wheat to corn. The authors attribute the improved performance of steers fed with a blend of grains to positive associative effects, possibly due to the differential degradation rates of the two grains (Kreikemeier et al., 1987).

Observed carcass characteristics in the current experiment are consistent with observations from our previous research where replacing one-third of DRC with DRR did not affect carcass characteristics (Rusche et al., 2020; Podversich et al., 2025). Moreover, in the study by Zhang et al. (2024a), replacing one-third of barley had minimal impact on carcass characteristics; however, in that study, liver abscess prevalence was increased with RG. The risk of ruminal acidosis with RG was characterized by a series of studies from the University of Saskatchewan, in which replacing barley with rye in beef diets negatively affected ruminal pH corresponding with greater incidence of liver abscesses (Zhang et al., 2024a, 2024b). The tendency for greater liver abscess incidence for DRR compared with HMR in the current experiment corresponds with differences observed in starch digestibility and with smaller GMD for DRR; however, we cannot definitively state that sub-acute acidosis was the causative pathway under the conditions of this experiment.

## CONCLUSION

Results of this study confirm our previous findings regarding the partial replacement of corn with hybrid rye in finishing cattle diets and further support our conclusion that rye grain can be included as part of the diet with little to no negative effects on performance or feed efficiency. Processing rye grain tended to improve feed efficiency compared with feeding whole rye grain. In this experiment, dry-rolling rye grain reduced DMI while maintaining similar ADG compared with using a hammer mill, resulting in greater dietary net energy values and a tendency for improved feed efficiency. This difference was likely the result of inadequate particle size reduction for hammer-milled rye under the conditions of this experiment.
